# Glycyrrhizic acid promotes sciatic nerves recovery in type 1 diabetic rats and protects Schwann cells from high glucose-induced cytotoxicity

**DOI:** 10.7555/JBR.36.20210198

**Published:** 2022-05-15

**Authors:** Min Shi, Xiangcheng Zhang, Ridong Zhang, Hong Zhang, Dalong Zhu, Xiao Han

**Affiliations:** 1 Key Laboratory of Human Functional Genomics of Jiangsu Province, Department of Biochemistry and Molecular Biology, Nanjing Medical University, Nanjing, Jiangsu 211166, China; 2 Department of Endocrinology, Nanjing Drum Tower Hospital Clinical College of Nanjing Medical University, Nanjing, Jiangsu 210008, China; 3 Department of Endocrinology, the Affiliated Huai'an No. 1 People's Hospital of Nanjing Medical University, Huai'an, Jiangsu 223300, China; 4 Department of Intensive Care Unit, the Affiliated Huai'an No. 1 People's Hospital of Nanjing Medical University, Huai'an, Jiangsu 223300, China

**Keywords:** diabetic peripheral neuropathy, glycyrrhizic acid, high-mobility group box-1, inflammation

## Abstract

The present study aims to investigate the therapeutic effect and mechanism of glycyrrhizic acid (GA) in diabetic peripheral neuropathy (DPN). GA significantly mitigated nerve conduction velocity (NCV) deficit and morphological abnormality and reduced high-mobility group box-1 (HMGB1) expression in the sciatic nerves of diabetic rats independent of blood glucose and body weight. Notably, GA alleviated the increase of HMGB1 and the decrease of cell viability in high glucose-stimulated RSC96 cells. Furthermore, GA obviously reduced the concentration of inflammatory cytokines in the sciatic nerves of diabetic rats and supernatants of high glucose-exposed RSC96 cells, then restored the decreased expression levels of nerve growth factor (NGF) and neuritin-1, and the increased expression levels of cleaved caspase-3 and neuron-specific enolase. Additionally, GA markedly inhibited receptor for advanced glycation end products (RAGE) expression, p38MAPK phosphorylation, and the nuclear translocation of NF-κBp65 in diabetic rats and high glucose-exposed RSC96 cells. The promotional effect of high glucose in RSC96 cells was diminished following *Hmgb1* siRNA treatment. Our findings indicate that GA may exert neuroprotection on DPN by suppressing HMGB1, which lead to extenuation of inflammation response, balance of NGF, neuritin-1 and caspase-3, as well as inactivation of RAGE/p38MAPK/NF-κBp65 signaling pathway.

## Introduction

Diabetic peripheral neuropathy (DPN), a well-known diabetic complication, affects at least 50% of diabetic patients^[1–2]^. It is generally agreed that DPN causes pain and discomfort in lower extremities, foot ulcerations, amputation, and reduced quality of life^[1–2]^. Although neurons have always been a major concern in the clinical and basic research of DPN, accumulating data are emphasizing the involvement of Schwann cells in DPN^[3–4]^. Schwann cells, serving as glial cells in the peripheral nervous system, can maintain peripheral nerve function by interacting with axons and blood vessels^[3,5]^. The substances released by Schwann cells can be internalized by neurons to stimulate axon regeneration, which opens a new dimension to our understanding of glial-axon regeneration during DPN^[5–6]^. Hyperglycemia and metabolic abnormalities either directly or indirectly contribute to morphological abnormalities and functional defects of Schwann cells^[7–8]^. It has been reported that dysfunctional Schwann cells provoke development of DPN, thus serving as a therapeutic target for DPN^[9]^.

Mounting evidence has demonstrated that chronic low-grade inflammation plays a critical role in the occurrence and progression of DPN^[3,10]^. The therapy against inflammatory factors, such as tumor necrosis factor α (TNF-α), intercellular adhesion molecule 1 (ICAM-1), and transforming growth factor-β, can relieve DPN in experiment animals, but the clinical benefit has not yet been proved^[10–11]^. Thus, there is an urgent need to discover a new therapeutic target that can control chronic inflammation process for preventing or treating DPN.

High-mobility group box-1 (HMGB1), an evolutionarily conserved non-histone chromatin-binding protein, plays an important role in regulating nuclear homeostasis^[12]^. HMGB1 is actively excreted or passively discharged into the extracellular regions in response to specific stimulations, such as infection, injury, and sterile inflammation^[12–13]^. Extracellular HMGB1 has also been shown to promote nuclear factor Kappa Bp65 (NF-κBp65)-dependent cytokine production and establish a proinflammatory vicious circle to sustain the immune process *via* its receptors, ultimately leading to tissue damage^[13–14]^. Emerging evidence has shown that HMGB1 is elevated in type 2 diabetes mellitus patients and experimental autoimmune encephalomyelitis (EAE) mice, a model of multiple sclerosis^[15–16]^. Furthermore, intravenous injection of anti-HMGB1 antibody into EAE mice prevents T cell infiltration into the central nervous system, inhibits systemic CD4^+^ T cell responses to myelin epitopes and disrupts the proinflammatory loop^[16]^. Overexpression of miR-193a can inhibit the HMGB1 production in the dorsal root of the lumbar spinal cord in streptozotocin (STZ)-induced diabetic mice, thereby reducing diabetic neuropathic pain^[17]^. HMGB1 blockade in the spinal cord also prevents tactile allodynia and thermal hyperalgesia of diabetic mice induced by STZ, which is related to the reduction of cytoplasmic HMGB1 expression and transport in the spinal dorsal root neurons (DRG)^[18].^ The above evidence suggests that HMGB1 is closely involved in pathogenesis of neuroinflammation and neurological diseases. These attributes make HMGB1 an attractive therapeutic target in disease treatment.

Glycyrrhizin acid (GA), a triterpene glycoside extracted from the roots of licorice plants, exhibits anti-inflammatory, anti-diabetic and anti-oxidant properties^[19]^. GA inhibits HMGB1 generation and mitogenic activities, and blocks its binding to its receptor^[20–21]^. The HMGB1-binding function of GA has been verified by using nuclear magnetic resonance and fluorescence^[19]^. It has been demonstrated that GA can ameliorate retinal vascular barrier dysfunction *via* the extracellular regulated protein kinases (ERK)/NF-κBp65 pathway deactivation by inhibiting HMGB1 activity and HMGB1-related inflammatory response in the diabetic retina^[22]^. Our previous study showed that GA prevents diabetes-induced renal lesion and inflammatory responses by suppressing receptor for advanced glycation end products (RAGE)/Toll-like receptor 4-related ERK and p38 mitogen-activated protein kinases (p38MAPK)/NF-κBp65 activation^[23]^. However, the potential effects and mechanism of GA in DPN remain unclear. Thus, this study aimed to investigate whether GA treatment could improve diabetic sciatic nerve injury by regulating HMGB1 and whether it exerted this effect by inhibiting HMGB1-mediated inflammatory pathways. The preventive effect and mechanism of GA in peripheral nerve abnormalities were explored both in *vivo* (type 1 diabetic rats) and*in vitro* (high glucose-exposed rat Schwann cells [RSC96]).

## Materials and methods

### Animal model establishment and glycyrrhizin acid treatment

Thirty specific pathogen free (SPF) Male Sprague-Dawley (SD) rats (8-week-old, weighing 180 to 200 g) were obtained from Shanghai Sippr-BK Laboratory Animal Co., Ltd. (China). All rats were housed under constant laboratory conditions with a 12-hour/12-hour light/dark cycle and allowed to acclimatize to the condition for 1 week before any intervention. The type 1 diabetic rat model was established by a single intraperitoneal injection of STZ (Sigma Aldrich, USA) at 60 mg/kg in 10 mmol/L sodium citrate buffer (pH 4.5)^[4,23]^. The normal control rats were injected with equal volumes of the citrate buffer. At one week after the first STZ injection, the blood glucose from the tail vein was measured. Rats with fasting blood glucose concentrations above or equal to 16.7 mmol/L were considered to indicate diabetes and used for further experiments. The animals were further randomly divided into three groups (*n*=10 per group): control group, diabetic group, and diabetic + GA group. The control and diabetic groups were treated with saline intragastrically, while diabetic + GA group received intragastrically GA (150 mg/[kg·day]; Santa Cruz Biotechnology, USA) shortly after the onset of diabetes throughout the experiment. The Fast Blood Glucose Monitoring System (Breeze 2, Bayer Healthcare, USA) was used to measure the levels of blood glucose each week throughout the experiment. All animal experiments were approved by the Ethics Committee of Animal Care of Nanjing Medical University (approved number: DW-P-2021-010-01) and followed the Guidelines for Animal Experiments of the Chinese Academy of Medical Sciences.

### Estimation of nerve conduction velocity

The DPN model was validated when nerve conduction velocity (NCV) was lower than 40 m/s. We estimated NCV in the sciatic nerve trunk of the right leg at the end of the 8^th^ week after diabetes, as described previously ( www.diacomp.org)^[4,10]^. Intraperitoneal anesthetization was performed on the rats with 10% chloral hydrate (350 mg/kg). Electrophysiological testing was implemented under standardized conditions and at a controlled temperature (room and animals). Briefly, motor nerve conduction velocity (MNCV) was determined by stimulating distally at the sciatic notch and the ankle *via* bipolar electrodes. MNCV was calculated by the distance between stimulating electrodes by the average latency difference. Sensory nerve conduction velocity (SNCV) was determined by stimulating the nerve distally at the ankle *via* bipolar electrodes. SNCV was calculated using the onset latency and distance.

### Sample collection and histopathological examination

After the NCV test, the bilateral sciatic nerve tissues were collected, fixed with 10% formaldehyde and processed for pathological and immunohistochemical staining. After dehydration, the samples were embedded in paraffin and dissected into 5-μm thickness on a rotary slicer (LEICA RM2135, Germany). The nerve damage and myelination status were measured by hematoxylin and eosin (H&E) staining with a light microscope (Nikon Eclipse 80i, Japan). The right sciatic nerve was immediately frozen in liquid nitrogen and retained at**−**80 °C until use.

### Cell culture and treatment

RSC96 cell line was obtained from the American Type Culture Collection (ATCC, USA), where they were tested and authenticated. These procedures included cross-species checks, DNA authentication, and quarantine. The role of hyperglycemia in the mylin-forming Schwann cells, the most abundant cells in the peripheral nervous system, was investigated. The cells were maintained at 37 °C in Dulbecco's Modified Eagle Medium (DMEM) (Thermo Fisher Scientific, USA) supplemented with 10% fetal bovine serum (FBS, Hyclone, USA), 100 U/mL penicillin, and 100 U/mL streptomycin under humidified conditions containing 95% air and 5% CO_2_. The cells were exposed to normal (5.6 mmol/L) or high glucose (25.0 mmol/L). Then, RSC96 cells incubated with high glucose medium were treated with various concentrations of GA (1, 10, and 100 μmol/L) and times (24 and 48 hours) to select vintage GA treatment for HMGB1 inhibition. To examine whether HMGB1 could contribute to the effect of inflammation on Schwann cell damage in high glucose condition, RSC96 cells were infected with siRNAs targeting *Hmgb1* (GCATCCTGGCTTATCCATT, RiboBio, China) to knock down HMGB1 according to the manufacturer's instructions. Two controls were used for these experiments, which included cells subjected to normal glucose (5.6 mmol/L) ambience and cells transfected with a non-targeting control pool.

### CCK-8 assay

The viability of RSC96 cells was measured using CCK-8 (Roche Diagnosis, Germany), following the manufacturer's instructions. To examine the impact of GA on cell proliferation, the cells were seeded into 96-well plates at a density of 5×10^3^ cells/well and allowed to adhere overnight. RSC96 cells were respectively incubated with normal glucose or high glucose medium for 24 hours. Moreover, cells were cultured in medium with 25 mmol/L high glucose and 10 μmol/L GA for 24 hours. The cells cultured in the medium with 5.6 mmol/L glucose served as the control. Cells transfected with control siRNA or *Hmgb1* siRNA were incubated into 96-well plates (5×10^3^ per well) with 10 μL of CCK-8 replenished to each well. After incubation for 2 hours at 37 °C at 5% CO_2_, the absorbance was appraised using a plate reader (ELx800, BioTek Instruments, Inc., USA) at 450 nm.

### Enzyme-linked immunosorbent assay

RSC96 cells were seeded into 48-well plates at a density of 1×10^4^ cells/well in DMEM and cultured until the next day. Following that, the culture medium was changed to 25.0 mmol/L DMEM supplemented with GA (0, 1, 10, and 100 μmol/L), before being collected at 24 and 48 hours. The medium was collected and centrifuged at 1500*g* at 4 °C for 10 minutes. HMGB1 was detected using rat HMGB1 enzyme-linked immunosorbent assay (ELISA) kit (R&D Systems, USA).

RSC96 cells were respectively incubated with normal glucose or high glucose medium for 24 hours. Again, a 10 μmol/L GA was applied to treat high glucose-cultured RSC96 for 24 hours before the medium was collected. RSC96 cells transfected with *Hmgb1* siRNA or siRNA control, respectively, were cultured in DMEM for another 24 hours. TNF-α, interleukin-1 β (IL-1β), interleukin-6 (IL-6), macrophage chemoattractant protein-1(MCP-1), and ICAM-1 in supernatants were measured by ELISA kit (R&D system) according to the manufacturer's instructions. A Multiskan FC Microplate Photometer (Thermo Scientific, USA) was used to detect the optical density at 450 nm. The standard curve was drawn using the data produced from the diluted standard solutions. The concentrations of HMGB1, TNF-α, IL-1β, IL-6, MCP-1, and ICAM-1 were calculated according to the standard curve.

### Real-time RT-PCR

Trizol reagent (Invitrogen Life Technologies, USA) was used to harvest total RNA from sciatic nerve and cells according to the manufacturer's instructions. The cDNA was synthesized using M-MLV reverse transcription kit (Invitrogen Life Technologies). Then, real-time RT-PCR analysis was carried out *via* a StepOnePlus Real-Time PCR System (Applied Biosystems, USA) with a SYBR Green qPCR kit (Takara Shuzo Co., Japan). Primers for the *Actb* and target genes are available in ***Table 1***. Calculation of relative quantification of mRNA was done by using the −2^ΔΔCt^ formula with *Actb* as an internal quantitative control.

**Table 1:** Primer sequences for real-time RT-PCR

Genes	Forward primer (5′-3′)	Reverse primer (5′-3′)
*Tnfa*	GTCTGTGCCTCAGCCTCTTC	TGGAACTGATGAGAGGGAGC
*Il1b*	CACCTCTCAAGCAGAGCACAGA	ACGGGTTCCATGGTGAAGTC
*Il6*	TCCAGTTGCCTTCTTGGGAC	GTACTCCAGAAGACCAGAGG
*Ccl2*	GTGCTGACCCCAATAAGGAA	TGAGGTGGTTGTGGAAAAGA
*Icam1*	AGGTATCCATCCATCCCACA	GCCACAGTTCTCAAAGCACA
*Ngf*	CTGGACCCAAGCTCACCTCA	GTGGATGAGCGCTTGCTCCT
*Nrn1*	GGGCGAAAGATATGTGGGAT	CGAGAGAGACACCAGGAGCA
*Nse*	GAACTATCCTGTGGTCTCC	CGACATTGGCTGTGAACTTG
*Actb*	CACCCGCGCGTACAACCTTC	CCCATACCCACCATCACACC

### Western blotting

The sciatic nerve samples and cells were lysed in a Western lysis buffer (30 mmol/L Tris-HCl, pH 7.5, 5 mmol/L EDTA, 1% Triton X-100, 250 mmol/L sucrose, 1 mmol/L Sodium vanadate, protease inhibitor cocktail and phosphatase inhibitor) and the lysate was centrifuged at 12 000 *g* for 20 minutes at 4 °C. The supernatants were collected. Equal amounts of protein (40 μg) underwent SDS-PAGE electrophoresis on 10% gel prior to the transfer onto PVDF membranes (Millipore, USA). Immunodetection was done using primary antibodies against cleaved caspase-3 (1:1000; Cell Signaling Technology, USA), HMGB1 (1:1000; Abcam, USA), RAGE (1:1000; Santa Cruz Biotechnology), NF-κBp65 subunit (1:2000; Cell Signaling Technology), p38MAPK (1:1500; Cell Signaling Technology), phospho-p38MAPK (Thr180/Tyr182; 1:2000; Cell Signaling Technology), ERK (1:1500; Cell Signaling Technology), p-ERK (Thr202/Tyr204; 1:2000; Cell Signaling Technology), JNK (1:1500; Cell Signaling Technology), p-JNK (Ser63/Ser73; 1:2000, Cell Signaling Technology), GAPDH (1:4000; Sigma Aldrich), nuclear protein Histone-H3 (1:1500; Santa Cruz Biotechnology) and neuron-specific enolase (NSE; 1:1000; Abcam). After incubated with primary antibodies at 4 °C overnight, the membranes were reacted with corresponding secondary antibodies for 1 hour at room temperature. The blots were washed, stained with Western Lightning Chemiluminescence Reagent (Millipore) and imaged using Chemi-DocXRSt systems (Bio-Rad laboratories Inc., USA). NE-PER Nuclear and Cytoplasmic Kits (Thermo Fisher Scientific) were used to prepare nuclear and cytoplasmic extracts.

### Statistical analysis

All statistical analyses were carried out using statistical analysis software (SPSS 18.0, SPSS Inc., USA). The data were expressed as mean±SD. Repeated measures ANOVA was used to analyze the differences in blood glucose and body weight for three groups. Differences in other indicators between the comparative groups were analyzed using Student's *t*-test and one-way ANOVA analysis. Statistical significance was considered when *P*<0.05.

## Results

### Glycyrrhizin acid did not significantly affect blood glucose levels and body weight in diabetic rats

**Figure 1:**
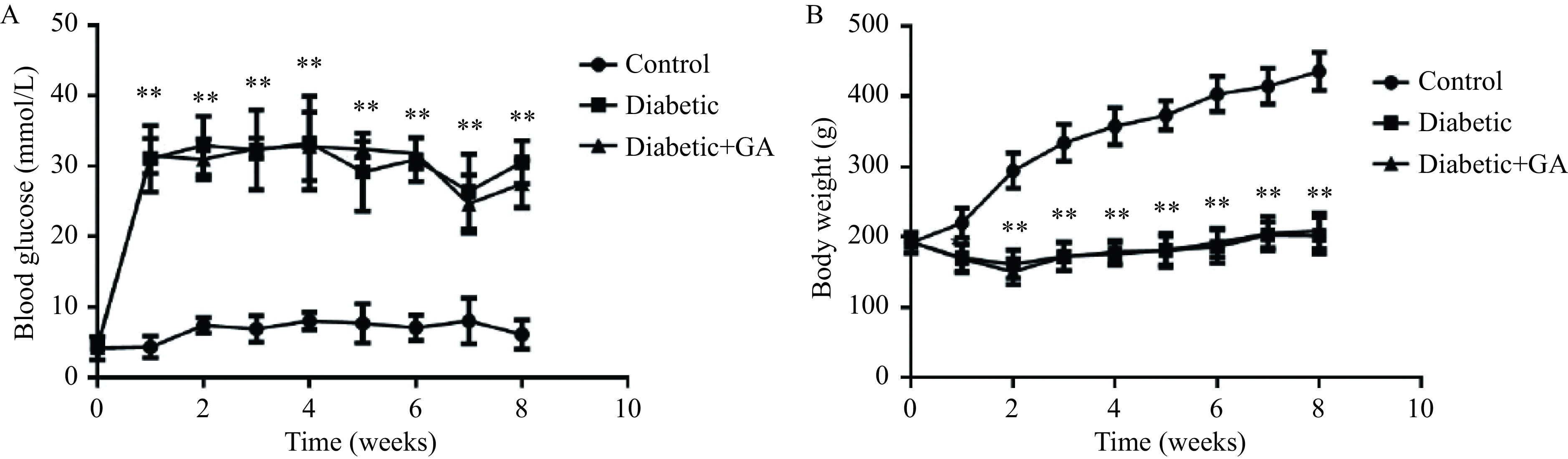
Effects of glycyrrhizin acid on blood glucose and body weight in diabetic rats.

Diabetic rats were treated with GA or saline for eight weeks. As shown in ***Fig. 1***, blood glucose levels were obviously increased (***Fig. 1A***) and body weight was significantly decreased in diabetic rats (***Fig. 1B***). After the 8-week GA administration, neither the hyperglycemia or weight loss were altered (***Fig. 1A*** and ***B***).

### Glycyrrhizin acid alleviated sciatic nerve injury in diabetic rats

**Figure 2:**
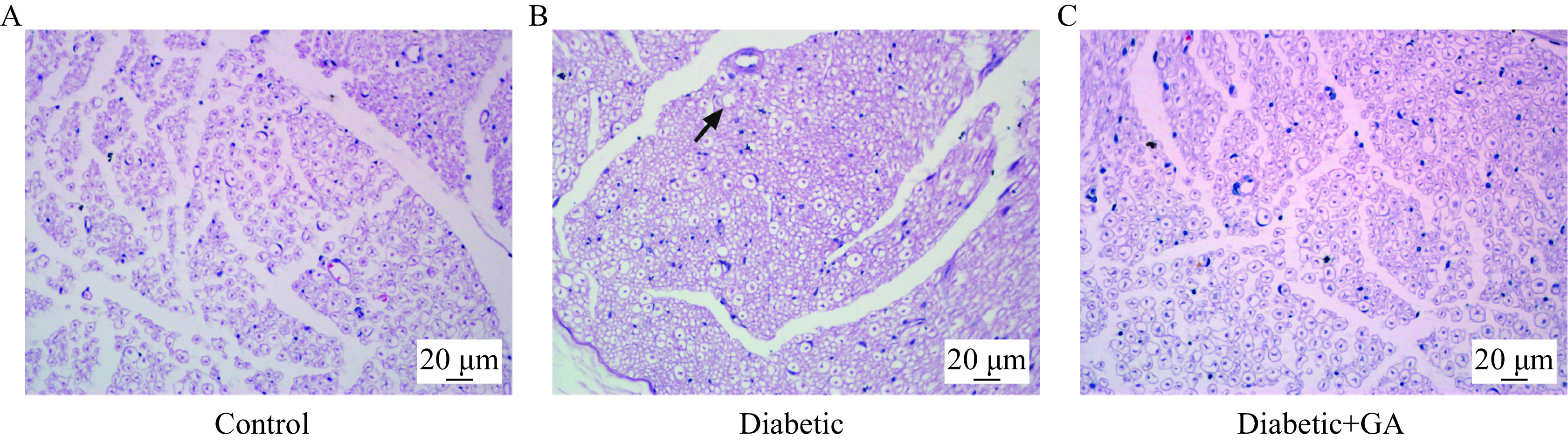
Effects of glycyrrhizin acid on histological morphology in the sciatic nerve.

To evaluate the effect of GA on sciatic nerve function in STZ-induced rats, MNCV and SNCV were measured. Diabetic rats displayed a marked decrease in MNCV and SNCV levels compared to the control rats. GA treatment partly reversed the diabetes-induced reductions in MNCV and SNCV (***Table 2***,*P*<0.05). In addition, the sciatic nerve fibers of control rats were arrayed regularly, each fiber was plump and myelin sheath was evenly stained (***Fig. 2A***). In contrast, the myelinated nerve fibers in diabetic rats presented irregularity, disruption, loose organization, and vacuolar-like defects in the myelin sheath (***Fig. 2B***), while this morphology of myelin was partly restored after GA treatment (***Fig. 2C***). Taken together, these results demonstrated that GA treatment ameliorated diabetes-induced sciatic nerve dysfunction independent of blood glucose and body weight.

**Table 2:** Evaluation of NCV of sciatic nerve in each experimental group

Groups	MNCV (m/s)	SNCV (m/s)	*n*
Control	43.76±4.97	46.38±6.27	10
Diabetic	34.77±5.12^*^	38.81±7.01^*^	6
Diabetic+GA	40.05±4.68^#^	41.46±5.49^#^	7
NCV was exmined after eight weeks intervention with glycyrrhizin acid in rats. Data are presented as mean±SD,*n*=6. Statistical analyses are performed by One-way ANOVA. ^*^P<0.05*vs.* Control group; ^#^*P*<0.05*vs.* Diabetic group. Rats of the Control and Diabetic groups received saline for eight weeks *via* gavage; rats of the Diabetic+GA group received glycyrrhizin acid (150 mg/[kg·day]) for eight weeks *via* gavage. GA: glycyrrhizin acid; NCV: nerve conduction velocity; MNCV: motor nerve conduction velocity; SNCV: sensory nerve conduction velocity.			

### Glycyrrhizin acid inhibited HMGB1 expression in the sciatic nerve of diabetic rats

**Figure 3:**
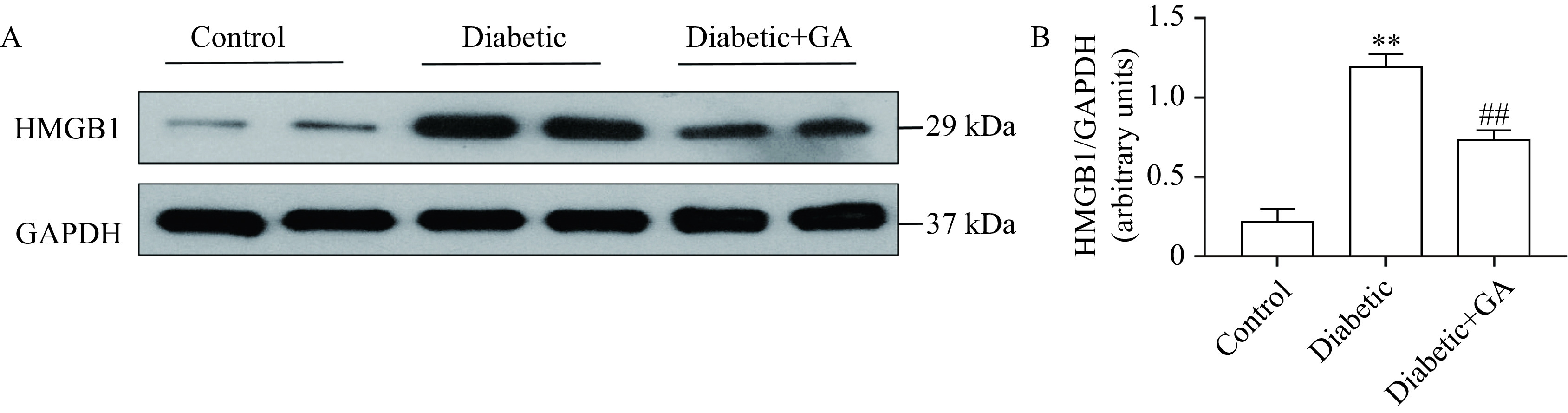
Effects of glycyrrhizin acid on HMGB1 expression in the sciatic nerve of diabetic rats.

Considering that GA was proven to be an inhibitor of HMGB1, we further explored HMGB1 expression in the sciatic nerve of rats with STZ-induced hyperglycemia. Western blotting analyses of the sciatic nerve tissues from diabetic rats revealed a notable increase in the HMGB1 protein expression, whereas an obvious decrease of HMGB1 expression was observed in GA-treated diabetic rats (***Fig. 3***).

### Glycyrrhizin acid modulated HMGB1 expression in Schwann cells under high glucose ambience

**Figure 4:**
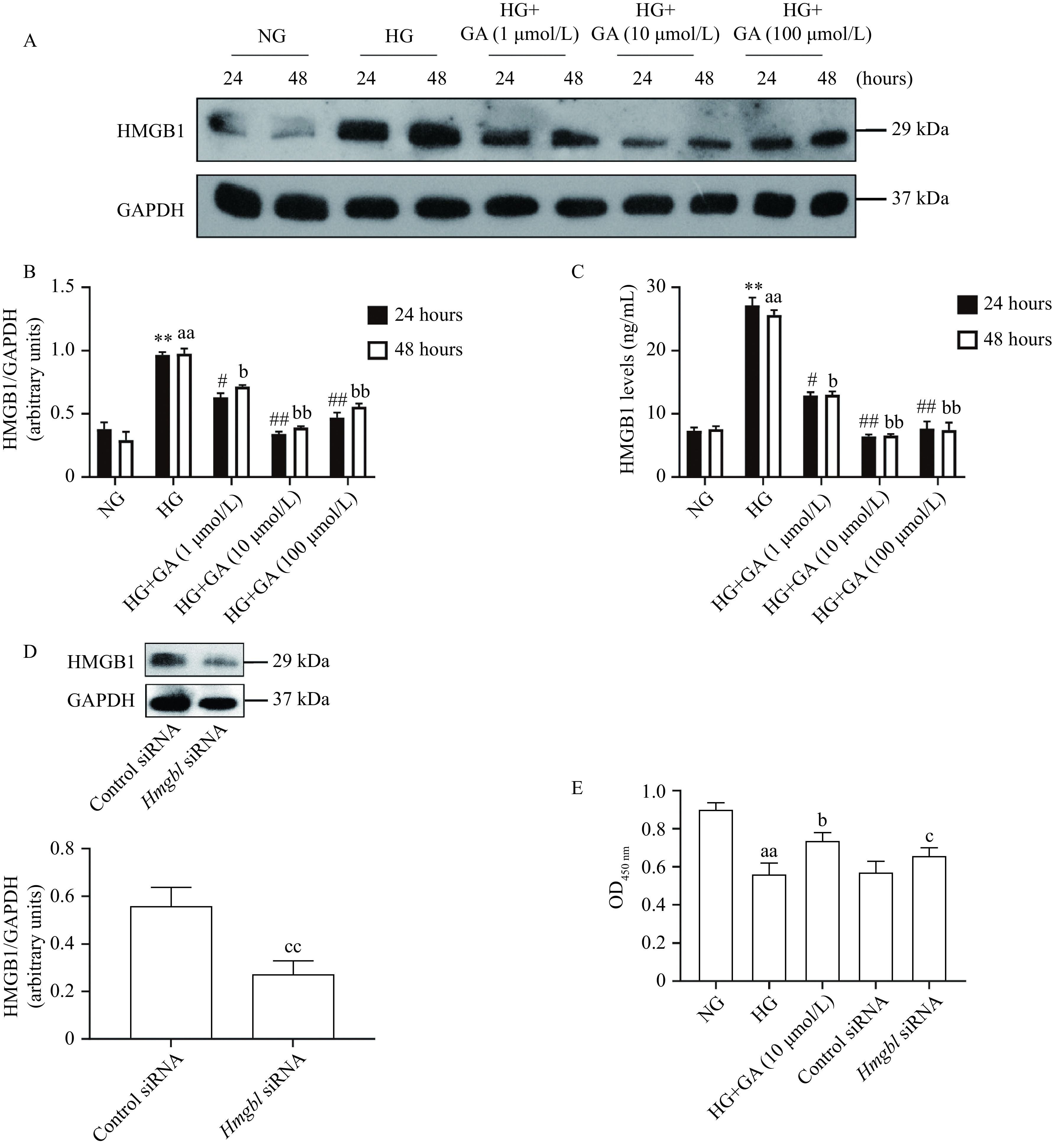
Effects of glycyrrhizin acid on HMGB1 in RSC96 cells.

We further detected the effect of GA on HMGB1 expression in RSC96 cells. Modulation of HMGB1 by high glucose ambience was investigated in RSC96 cells by using Western blotting and ELISA analyses. RSC96 cells were exposed to various glucose concentrations (5.6 and 25.0 mmol/L) and at different times (24 and 48 hours). HMGB1 expression in RSC96 cells exposed to a higher glucose concentration at 24 and 48 hours was much higher than that exposed to a lower concentration. A dose-dependent increase in its intensity was observed with the exposure to glucose (***Fig. 4A*** and ***B***). Then, the secreted HMGB1 protein level from culture medium was examined. In line with the results of Western blotting, the ELISA analyses revealed a dose-dependent increase in the secretion of HMGB1 in cells exposed to high glucose ambience (***Fig. 4C***). After incubation with GA at 10 and 100 μmol/L for 24 hours, the HMGB1 levels in the supernatants of high glucose (25.0 mmol/L)-treated cells were noticeably restrained. Although the data showed that HG-induced HMGB1 levels were effectively reduced by both 10 and 100 μmol/L of GA, the dose of 10 μmol/L was preferred to further study.

To examine the knock-down efficiency of HMGB1 gene in RSC96 cells, protein expression was measured by Western blotting analysis (***Fig. 4D***). HMGB1 protein expression declined in HMGB1 knock-downed RSC96 cells. Exposure of RSC96 cells to 25.0 mmol/L high glucose led to a decreased cell vitality. Raised HMGB1 expression inhibited cell vitality (***Fig. 4E***). We thus supposed the cell vitality was associated with HMGB1 expression. Moreover, increased protein synthesis was dampened by GA in cells subjected to high glucose ambience. However, these effects of high glucose ambience were negated in cells transfected with *Hmgb1*-siRNA (***Fig. 4E***). Transfection of control siRNA did not influence the protein synthesis or cell vitality. These findings suggested that HMGB1 inhibition mediated GA-improved cell vitality in high glucose-cultured RSC96 cells.

### Glycyrrhizin acid attenuated inflammation response in diabetic rats and high glucose-exposed RSC96 cells

**Figure 5:**
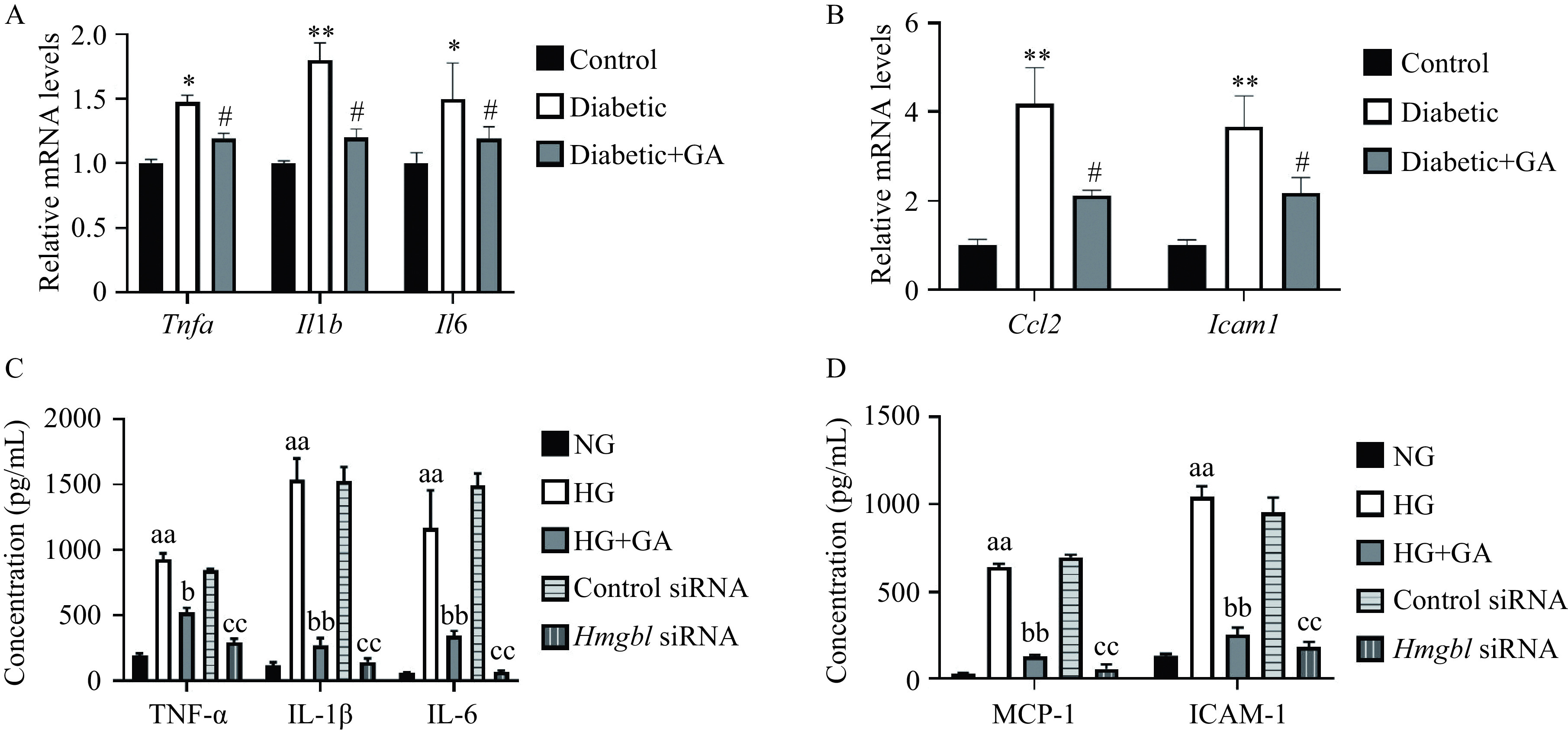
Effects of HMGB1 inhibition on inflammatory factors.

Considering that HMGB1 could regulate inflammation-induced tissue damage, we surmised that HMGB1 inhibition could mediate GA-normalized inflammation in diabetic rats. The concentrations of inflammatory factors in the sciatic nerve were measured. The expression of *Tnfα*, *Il1b*, and *Il6* in the sciatic nerve was evidently raised in the diabetic group in comparison to the control group and restored by GA intervention as determined by real-time RT-PCR and Western blotting (***Fig. 5A*** and ***Supplementary Fig. 1*** [available online]). In addition, *Ccl2* and *Icam1* expression was also substantially elevated in diabetic rats, while GA supplement reversed STZ-induced elevation in *Ccl2* and *Icam1* in the diabetic rats (***Fig. 5B*** and ***Supplementary Fig. 1***).

Subsequently, we determined the influence of GA intervention on inflammatory factor generation in high glucose-cultured RSC96 cells by ELISA and Western blotting assay. As shown in ***Fig. 5C*** and ***D***, GA also obviously reduced the content of TNF-α, IL-1β, IL-6, MCP-1, and ICAM-1 in the supernatants of HG-challenged RSC96 cells. The inhibitory effect of GA on inflammation response in cell experiment was consistent with that of animal experiment. We also found that the promotional effect of HG on the levels of TNF-α, IL-1β, IL-6, MCP-1, and ICAM-1 in the supernatants of RSC96 cells was notably attenuated by *Hmgb1* siRNA (***Fig. 5C***, ***D***, and ***Supplementary Fig. 2*** [available online]). Collectively, these data demonstrated that HMGB1 inhibition-mediated alleviation of pro-inflammatory factors in Schwann cells played protective roles in DPN.

### Glycyrrhizin acid increased nerve growth factor and neuritin-1 expression and inhibited the cleaved caspase-3 formation

**Figure 6:**
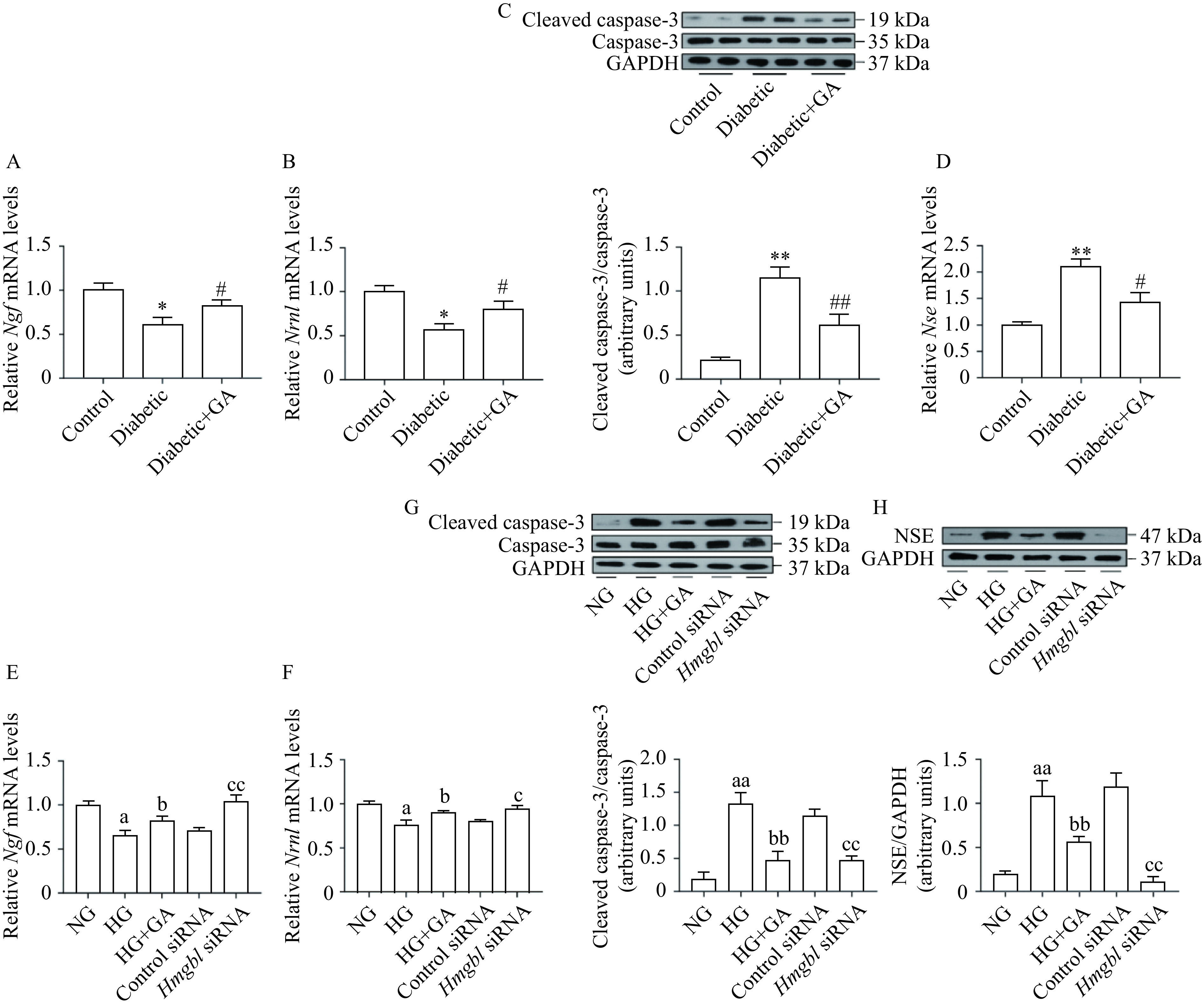
Effects of HMGB1 inhibition on alternation of neurotrophic factors, caspase-3 activity, and NSE expression.

In addition to forming myelin sheaths around axons, Schwann cells also actively secrete neurotrophic factors for neuron survival, regeneration, and activities^[8]^. Considering the influence of neurotrophic factor on peripheral nerve function, we explored the potential effect of GA on NGF and neuritin-1, which were proven to be the regulators of DPN in the previous studies^[24–26]^. Here, we found that the mRNA expression of *Ngf* and *Nrn1* was decreased, and the expression level of cleaved caspase-3 was considerably up-regulated in sciatic nerve tissue of diabetic rats and HG-exposed RSC96 cells (***Fig. 6A***–***C***). However, GA treatment greatly weakened these effects of high glucose ambience. NSE mediated formation of phosphoenolpyruvate, which may be elevated in and serviced as an indicative of DPN^[27–28]^. The addition of GA reversed the diabetes-provoked *Nse* production (***Fig. 6D***). As seen in ***Fig. 6E***–***H***, the inhibition of HMGB1 by *Hmgb1* siRNA effectively avoided the decreased NGF and neuritin-1 gene expression and the increased cleaved caspase-3 and NSE in hyperglycemic RSC96 cells. These data suggested that the balance between NGF, neuritin-1 and caspase-3 was regulated by HMGB1 blockade, which may be a potential therapy for diabetic sciatic nerve injury

### Glycyrrhizin acid inhibited the activation of RAGE/p38MAPK/NF-κBp65 signaling pathway

**Figure 7:**
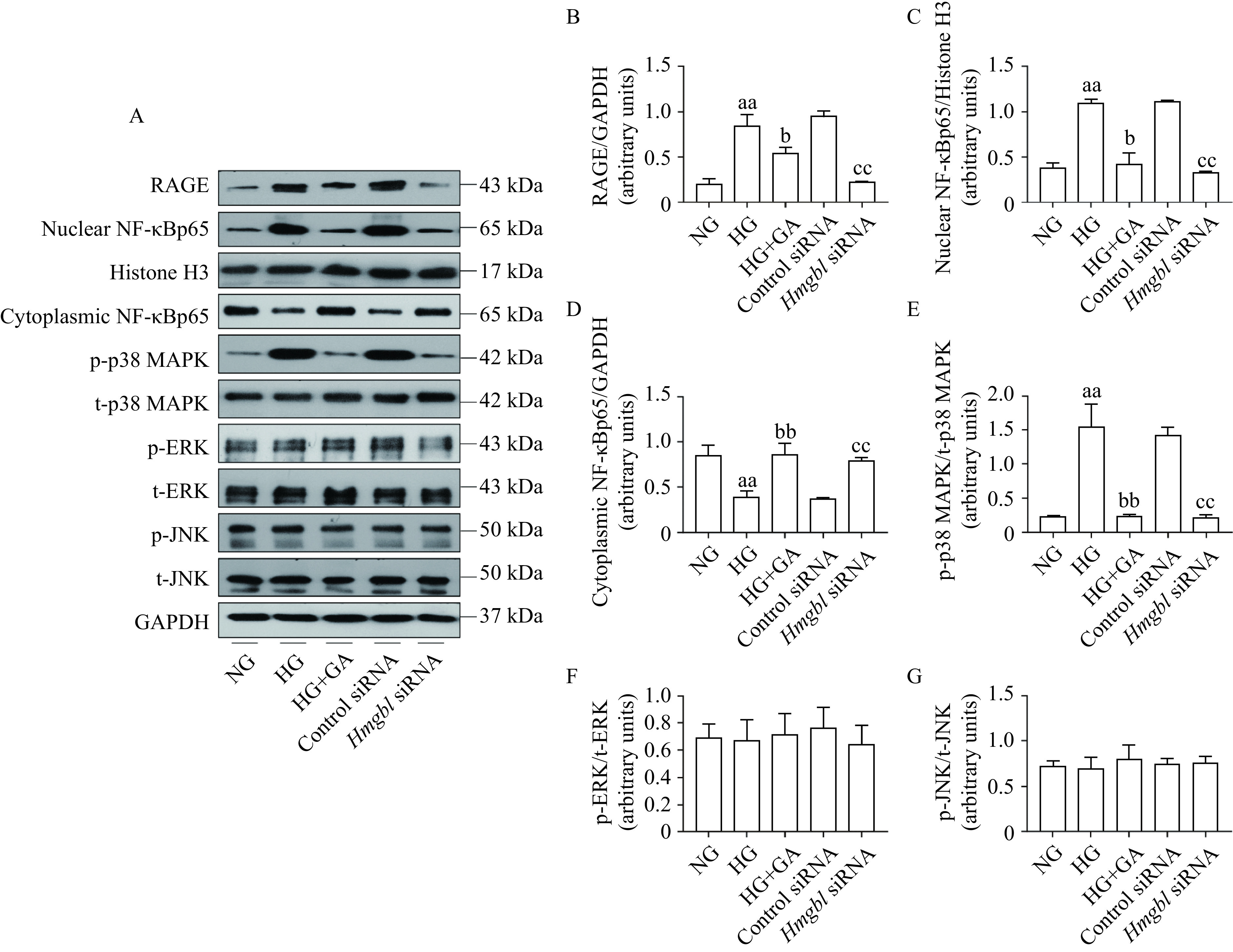
Effects of HMGB1 inhibition on characterization of RAGE, NF-κBp65, and MAPKs protein expression in the RSC96 cells.

To further figure out the mechanism involved in GA-improved nerve injury, Western blotting was applied. The RAGE protein level was presented in ***Supplementary Fig. 3*** (available online) and ***Fig. 7***. The overexpression of RAGE in the sciatic nerve of diabetic rats and HG-exposed RSC96 cells was markedly inhibited by GA treatment (***>Supplementary Fig. 3A*** and ***B***, ***Fig. 7A*** and ***B***). The results indicated that RAGE expression was dramatically inhibited in RSC96 cells due to *Hmgb1* siRNA (***Fig. 7A*** and ***B***). Moreover, an increase in nuclear NF-κBp65 and a decrease in cytoplasmic NF-κBp65 were observed in the sciatic nerve of diabetic rats and HG-exposed RSC96 cells (***Supplementary Fig. 3A***, ***C***, and ***D***, ***Fig. 7A***, ***C***, and ***D***). However, the inhibition of HMGB1 with GA treatment reversed the effect by suppressing the translocation of NF-κBp65 under diabetic conditions (***Fig. 7A**, **C***, and ***D***). Furthermore, the phosphorylation levels of MAPKs, upstream signaling molecules of NF-κBp65 were examined. The phosphorylation level of p38MAPK was remarkably up-regulated in the diabetic rats and HG-cultured RSC96 cells, which was strikingly restored by GA (***Fig. 7A*** and ***E***). The phosphorylation levels and expression of ERK and JNK remained unchanged among the three groups (***Fig. 7A***, ***F***, and ***G***). *Hmgb1* siRNA in HG-exposed RSC96 cells led to a decrease in nuclear NF-κBp65 as well as reduced phosphorylation of p38MAPK (***Fig. 7A***, ***C***, and ***D***). Based on the above observations, an inverse association between GA and the proteins HMGB1, RAGE, p38MAPK and NF-κBp65 suggested that GA potentially targeted HMGB1 and its signal pathway in the sciatic nerve tissues, which might contribute to improvement of peripheral nerve function.

## Discussion

DPN is an intractable complication of diabetes, as an effective treatment has not been validated yet. Inflammation has been recognized to play a vital role in the pathogenesis of DPN^[11]^. This study provided the first evidence that GA treatment suppresses inflammation and the subsequent neurotrophic disorders by inhibiting HMGB1 generation in the sciatic nerve of diabetic rats and high-glucose-treated RSC96 Schwann cells.

Persistent hyperglycemia causes functional and structural damage to sciatic nerves, including axonal loss, axonal regeneration and myelin sheath abnormality^[29]^. In this study, GA was found to improve sciatic nerve function by restoring MNCV and SNCV. The results of HE staining analysis suggested GA ameliorated the sciatic nerve histopathology of diabetic rats. Moreover, GA treatment repaired sciatic nerve injury in diabetic rats independence of blood glucose and body weight. GA has been proved to have a variety of pharmacological effects by binding selectively to HMGB1 protein released extra-cellularly and inhibiting its cytokine activities through a scavenger mechanism on the protein accumulation^[21]^. In this present study, we found that GA decreased HMGB1 expression in the sciatic nerves of diabetic rats, thus improving nerve damage.

RSC96 cells model is widely used to investigate the characteristics of diabetic damage, screen effective drugs for the treatment of DPN, and study the mechanism of DPN^[8,30]^. We found that GA treatment down-regulated HMGB1 expression in high glucose-cultured RSC96 cells, accompanied with the recovery of cell vitality. Hyperglycemia-related cell vitality decline could be mediated by HMGB1, which was confirmed by siRNA technology. HMGB1, known as a dynamic protein, regulates the production of a variety of pro-inflammatory cytokines and the proliferation and activation of many inflammatory cells^[31–32]^. Macrophages can be induced by HMGB1 to polarize towards a pro- (M1) or anti-inflammatory (M2) phenotype^[33]^. Extracellular HMGB1 promotes the development of macrophages with a neurotoxic phenotype, which is consistent with classically activated inflammatory M1 macrophages. Neurotoxic macrophage functions elicited by HMGB1 contribute to secondary injury after spinal cord injury^[34]^. It has been reported HMGB1 inhibition could attenuate traumatic brain injury by inhibiting M1 phenotype while inducing M2 phenotype activation of microglia/macrophages^[35]^. HMGB1 is involved in the pathological lesion affecting various organs including the brain, heart, retina, and kidney *via* the modulation of inflammation and immune responses^[21–23]^. Blocking excessive extracellular HMGB1 paves a new way for inflammatory diseases, and specific antagonists targeting HMGB1 have achieved encouraging results in many experimental models of infectious and aseptic inflammation^[31,36]^. GA extracted from licorice is a natural inhibitor of HMGB1 with minimal side effects. Previous studies have revealed that HMGB1 inhibitor GA treatment protects against kidney lesions induced by diabetes, which is related to the reduced expression of TNF-α, 1L-6, IL-1β, MCP-1, and ICAM-1 in the kidney^[23]^. Yan *et al* confirmed that GA could significantly improve brain edema after cerebral ischemia-reperfusion and reduce the area of cerebral infarction, with decreased secretion of inflammatory cytokines in serum and brain tissue, including IL-1β, IL-6, and TNF-α^[37]^. In our current study, we confirmed the presence and overexpression of inflammatory factors in the sciatic nerves of diabetic rats and high glucose-treated RSC96 cells. Furthermore, we inhibited HMGB1 activity and observed the suppression of inflammatory factors related to hyperglycemia. Taken together, these results indicate that GA can suppress HMGB1 and it-interrelated inflammation *in vivo* and *in vitro*.

Neurotrophic factor deficits in Schwann cells are generally considered to contribute to DPN, and these deficits may result in disorganization and loss of myelin^[3–4,25]^. Among these factors, NGF promotes axonal growth of the sensory neuron by activating high-affinity tyrosine kinase (TrkA) or low-affinity p75 receptors^[25]^. NGF induces neuritin-1 expression (a new potential neurotrophic factor) in sensory neurons in a time- and dose-dependent manner *in vitro*, while inhibition of neuritin-1 abolishes NGF-mediated neurite outgrowth^[25]^. NGF contents in sciatic nerves and the expression of NGF receptor TrkA in DRG decrease in type 1 diabetic rats, whereas active caspase-3 expression increases^[38]^. NGF removes active caspase-3 by reducing the level of the p17 subunit in PC12 cells undergoing apoptosis by various cytotoxins^[39]^. Reduced cell neuritin-1 expression, increased apoptosis rates, increased caspase-3 activities and progressively reduced viability are observed in Schwann cells isolated from diabetic rats and cultured in high glucose^[4]^. *In vitro*, a decrease of neuritin-1 expression is related to apoptosis, whereas survivability and functions of Schwann cells are improved by exogenous neuritin-1 treatment with decreased caspase-3 activities^[4]^. Previous studies have suggested that NGF and neuritin-1 are down-regulated and associated with apoptosis of SCs exposed to high glucose milieu^[4,30]^. HMGB1 is a key factor in the regulation of neuroinflammation and hippocampal neuronal apoptosis in type 2 diabetic mice exposed to intermittent hypoxia^[40]^. HMGB1 accumulation activates complex signaling network for apoptosis. MiR-34α promotes the retinal cell apoptosis in DR rats through up-regulating the HMGB1 expression^[41]^. In this study, we first discovered that high glucose triggers neuroinflammation directly. Interestingly, the increase of HMGB1 is detected in both sciatic nerves of diabetic rats and high glucose-cultured Schwann cells. Then, hyperglycemia induces significant downregulation of the gene expression of Ngf and nrn1, whereas the expression of cleaved caspase-3 and NSE is significantly upregulated. Moreover, reverse parallel alterations between proinflammatory cytokines and neurotrophic factors are apparently observed *in* *vivo* and *in* *vitro*. Conversely, *Hmgb1* siRNA improves high glucose-mediated vitality, accompanied by elevated neurotrophin, lessened caspase-3, and decreased secretion of pro-inflammatory cytokines. The mechanism underlying the negative impact of chronic inflammation on neurodegeneration in diabetes is the reduction of neurotrophic factors and activation of caspase-3 by HMGB1-related proinflammatory cytokines, which is confirmed by *Hmgb1* gene knockout. Taken together, it has been verified that the inhibition of HMGB1 mediates GA-resumed neurotrophin and apoptosis-interrelated protein *via* affecting inflammation.

Furthermore, we explored the potential mechanism involved in GA neuroprotection effect. It has been well documented that RAGE, a main receptor of HMGB1, is required for HMGB1-induced inflammation, immunity, cell migration and regeneration^[42]^. HMGB1 induces the inflammatory mediators and its own release by binding to RAGE, forming a feedback loop^[31,36]^. The importance of HMGB1-RAGE-NF-κBp65 axis in the inflammatory cascade is demonstrated in diabetic microangiopathy and neurodegeneration^[22–23,43]^. As a key regulator of oxidative stress, NF-κBp65 can regulate the gene transcription of proteins in inflammation^[22–23]^. Our results showed that RSC96 cells incubation with high glucose led to RAGE and nuclear NF-κBp65 generation. In consistency with *Hmgb1* siRNA, GA supplement could also protect against RSC96 cell injury caused by HMGB1 synthesis *via* reducing proinflammatory cytokine generation and inhibiting RAGE/nuclear NF-κBp65 protein expression. MAPKs, the upstream of NF-κBp65, are closely associated with the pathogenic progress of DPN^[24–44]^. In the dorsal root ganglia of STZ-induced type 1 diabetic rats, ERK and p38MAPK were activated at eight weeks, while JNK was activated at 12 weeks^[44]^. p38MAPK, a negative regulator of Schwann cells differentiation and myelination, is activated in Schwann cells cultured in high glucose^[45–46]^. It has been confirmed that HMGB1 can promote the phosphorylation of p38MAPK and ERK1/2 in macrophages, and enhance lipopolysaccharide-stimulated pro-inflammatory cytokine TNF-α secretion^[47]^. GA can inhibit the HMGB1-induced migration of monocytes by blocking the activation of MAPK/ERK/NF-κBp65 signal pathways and the consequent MCP-1 expression^[48]^. GA is proved to exert a strong anti-inflammatory effect on diabetic retina and kidney damage *via* inhibiting MAPKs signaling pathway^[22,23]^. *In vitro* studies of glucose-stimulated RSC96 cells showed that the enhanced nuclear NF-κBp65 expression was apparently dependent on the upregulated p38MAPK signaling, and was weakened by stimulation of Schwann cells with GA and *Hmgb1* siRNA. HMGB1 has been shown to activate the MAPKs pathway, which is closely related to nerve formation and inflammation^[49–50]^. We found that GA or *Hmgb1* siRNA effectively reduces the expression of HMGB1 and RAGE in Schwann cells, together with inhibited NF-κBp65 activity and p38MAPK phosphorylation, which might contribute to slowing secretion of inflammation-related factors. The protective effect of GA on high glucose-provoked Schwann cell injury may be related to the suppression of inflammation triggered by HMGB1 and activation of RAGE, p38MAPK, and NF-κBp65.

Whether serum HMGB1 levels are correlated with pathological changes in the sciatic nerve warrants further investigation. Furthermore, it would be helpful to build diabetic RAGE^–/–^ mouse models and specific inhibitors for receptor and signal molecules based on the current data, so as to provide further evidence that the HMGB1/RAGE axis is related to the glial, neural and vascular degeneration in DPN.

Our study shows that HMGB1 released from Schwann cells in diabetes milieu may promote neuroinflammation in DPN *via* excessive activation of RAGE/p38MAPK/NF-κBp65 pathways. The neuro-protective effect of GA in DPN can be achieved by blocking HMGB1 and the resultant inflammation, together with neurotrophins deficiency and apoptosis-related proteins expression, which is related to the inhibition of RAGE-p38MAPK-NF-κBp65 pathway. These findings suggest that GA intervention may be an advantageous therapeutic approach for DPN.
